# Efficacy of femtosecond lasers for application of acupuncture therapy

**DOI:** 10.1007/s10103-016-2124-3

**Published:** 2016-12-10

**Authors:** Mika Ohta, Yoichiroh Hosokawa, Naoya Hatano, Aki Sugano, Akihiko Ito, Yutaka Takaoka

**Affiliations:** 1 Division of Medical Informatics and Bioinformatics, Kobe University Hospital, Kobe, 650-0017 Japan; 2 Genome Science Research Unit, Life Science Research Center, Kobe Tokiwa University, Kobe, 653-0838 Japan; 3 Graduate School of Materials Science, Nara Institute of Science and Technology, Nara, 630-0912 Japan; 4 The Integrated Center for Mass Spectrometry, Kobe University Graduate School of Medicine, Kobe, 650-0017 Japan; 5 Department of Pathology, Kindai University Faculty of Medicine, Osaka, 589-8511 Japan

**Keywords:** Femtosecond laser, Acupuncture therapy, Skeletal muscle, Myostatin, mTOR, p70S6K

## Abstract

Acupuncture treatment utilizes the stimulation of metal acupuncture needles that are manually inserted into a living body. In the last decades, laser light has been used as an alternative to needles to stimulate acupuncture points. We previously reported suppression of myostatin (Mstn) gene expression in skeletal muscle by means of femtosecond laser (FL) irradiation, after electroacupuncture, in which acupuncture needles are stimulated with a low-frequency microcurrent. The purpose of the study here was to investigate the efficacy of FL irradiation in mouse skeletal muscle with regard to protein synthesis. After irradiation of the hindlimbs, we first analyzed Mstn gene expression and Mstn protein level in the skeletal muscle. We then evaluated phosphorylation of the mammalian target of rapamycin (mTOR) and its downstream target 70-kDa ribosomal protein S6 kinase (p70S6K). The results showed that FL irradiation significantly reduced the amount of Mstn protein and enhanced the phosphorylation of p70S6K in of the mTOR/S6K signaling pathway. We suggest that FL irradiation activated the protein synthetic pathway in the skeletal muscle. In conclusion, we determined that FL irradiation can serve as an alternative for acupuncture needles and has the potential of being a new non-invasive acupuncture treatment of skeletal muscle.

## Introduction

Low-level lasers have been used in laser acupuncture, and their analgesic effects have been reported [1, 2]. Low-level lasers normally generate heat during irradiation, whereas heat generation at femtosecond laser (FL) focal spots is suppressed because of effective conversion of energy to kinetic waves [3]. In our previous FL study, we did not detect heat denaturation in animal tissues after irradiation [4]. The damage caused by the irradiation was observed in the epidermis and dermis, and the diameter of the damaged area and its depth were approximately 400 and 1100 μm, respectively. Such effects on animal tissue are similar to those of acupuncture treatment with acupuncture needles. Therefore, we believed that the FL was a good candidate for laser acupuncture.

We also elucidated expression of the *Mstn* gene after electroacupuncture (EA) treatment, which is one type of acupuncture stimulation [5]. Mstn, a member of the transforming growth factor-β superfamily, is a potent negative regulator of skeletal muscle mass [6]. Mstn has inhibited activation of satellite cells in skeletal muscle [7]. Mstn inactivation induced skeletal muscle hypertrophy in humans and mice [8, 9]. Our previous study indicated that EA treatment suppressed *Mstn* expression, which led to a satellite cell-related proliferative reaction and repair in skeletal muscle [5]. Other results showed that EA-induced *Mstn* gene suppression may help prevent muscle atrophy in mice [10]. We subsequently found suppression of expression of the *Mstn* gene in mouse skeletal muscle after FL irradiation, just as with EA [4, 11].

On the basis of the results of our previous studies, we expected activation of the protein synthetic pathway because Mstn inhibits activation of the Akt/mTOR pathway [12, 13]. The Akt/mTOR pathway regulates protein synthesis and is upregulated during skeletal muscle hypertrophy. The mTOR/S6K pathway includes the 70-kDa ribosomal protein S6 kinase (p70S6K) protein downstream, and the protein functions in the mTOR/S6K signaling pathway, which regulates protein synthesis and cell growth [14].

This study aimed to elucidate the efficacy of FL irradiation of mouse skeletal muscle. We investigated *Mstn* gene expression, the amount of Mstn protein, and phosphorylation of mTOR and p70S6K after irradiation.

## Materials and methods

### Animals and treatment

**Fig. 1:**
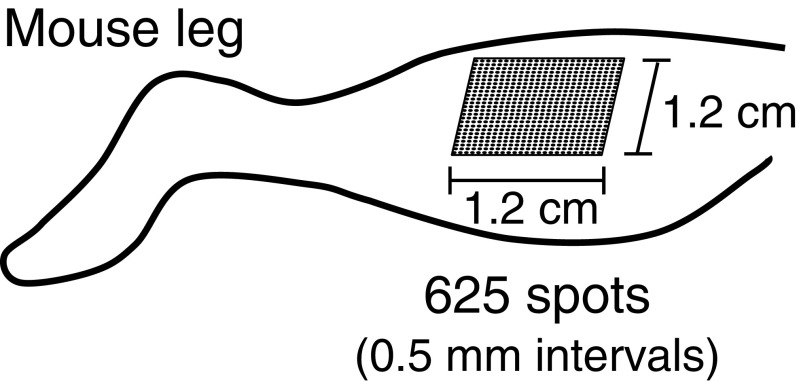
Schematic drawing of FL spots of the mouse hind leg

C57BL/6J male mice, 8 weeks old, were purchased from Charles River Laboratories, Yokohama, Japan. We divided the mice into three groups: controls, *n* = 5; acupuncture (ACP) stimulation, *n* = 6; and FL, *n* = 6. After anesthesia to all the three groups, hair on the hind legs of FL mice was removed by using hair removal cream, and FL treatment focused on the lower legs. A single-shot FL pulse with an energy of 300 μJ/pulse was picked up from a laser pulse train generated by a regeneratively amplified FL system (Spectra Physics, Hurricane, 800 nm, 150 fs, 20 Hz) and was focused on the target with a convex lens with a focal length of 150 mm. The laser focal position was sequentially changed after the laser shooting by controlling a motorized stage [4]. The laser irradiation spots, a total of 625, were made at regular 0.5-mm intervals on the 1.2-cm-square area (Fig. 1). For ACP stimulation, we used stainless-steel acupuncture needles (40 mm long and 0.16 mm in diameter; Seirin, Shizuoka, Japan). We manually inserted the needles into mice hind legs as to reach to the skeletal muscle (around 5 mm depth) and then removed them immediately, for a total of 182 places on the similar area to that of FL group. At 5 h after the treatments of all groups, muscle tissue samples were collected and stored at −80 °C until used. All mice were treated according to the Standards Relating to the Care and Management of Experimental Animals (Ministry of the Environment, Tokyo, Japan). This study was approved by the Committee for Safe Handling of Living Modified Organisms at Nara Institute of Science and Technology (Permission number 1132) and was carried out according to the guidelines of the committee.

### Real-time PCR analysis

We extracted total RNA from skeletal muscle by using the TRIzol reagent (ThermoFisher, Waltham, MA). cDNA was synthesized using the SuperScript First-Strand Synthesis System for RT-PCR (ThermoFisher, Waltham, MA). Real-time PCR was performed by using TaqMan Fast Advanced Master Mix and TaqMan Gene Expression Assays for Mstn and α-tubulin, according to the manufacturer’s instructions (ThermoFisher, Waltham, MA). All data were normalized to α-tubulin expression.

### Western blotting analysis

Triceps surae muscles from the mice were homogenized in PhosphoSafe reagent (Novagen, Madison, WI) that included a supplement protease inhibitor cocktail tablet (Roche, Indianapolis, IN). After homogenization, the samples were centrifuged at 12,000×*g* for 25 min at 4 °C. The Micro BCA Protein Assay Kit (Pierce, Rockford, IL) was used to determine the amount of protein, with bovine serum albumin as the standard. Protein samples were analyzed by using sodium dodecyl sulfate-polyacrylamide gel electrophoresis, after which they were transferred to polyvinylidene fluoride membranes (Amersham, Piscataway, NJ). Separated polypeptides were analyzed via Western blotting as previously described [11]. Primary antibodies included polyclonal anti-Mstn antibody (Millipore, Billerica, MA) and α-tubulin loading control (Abcam, Cambridge, MA). We also used primary antibodies against mTOR, phospho-mTOR (Ser 2448), p70S6K, and phospho-p70S6K (Thr 389), all of which were obtained from Cell Signaling Technology, Danvers, MA. Goat anti-rabbit IgG horseradish peroxidase conjugate (Cell Signaling Technology, Danvers, MA) was used for secondary detection. ECL Prime Western Blotting Detection Reagent (GE Healthcare, Buckinghamshire, Great Britain) was used to detect immunoreactive bands. All blots were scanned, and protein bands were quantified via ImageJ software (http://rsbweb.nih.gov/ij/). The relative protein levels of Mstn were calculated by means of comparison with the control (α-tubulin). Phosphorylation levels were calculated as the ratios of phospho-mTOR, phospho-p70S6K to total mTOR, p70S6K, respectively.

### Statistical analysis

All values are means ± standard deviations. Significance of real-time PCR data were examined using one-way ANOVA. Western blotting data were evaluated by using unpaired Student’s *t* test. The differences were considered statistically significant at *P* values of <0.05.

## Results

**Fig. 2:**
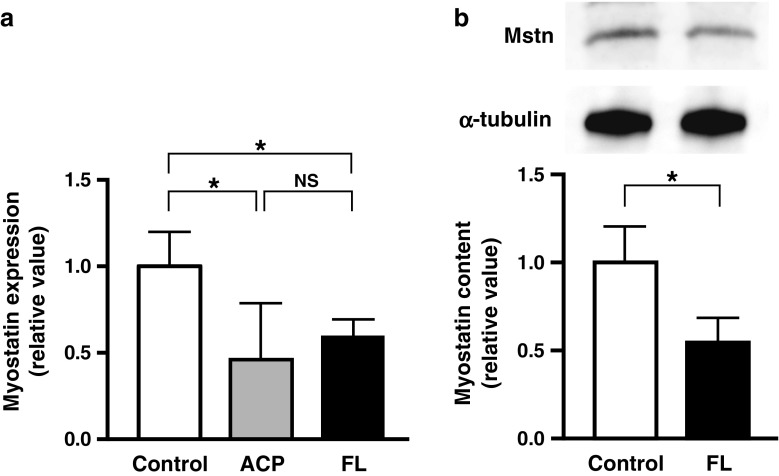
Analysis of *Mstn* gene expression and the amount of Mstn protein after FL irradiation. *Mstn* gene expression after FL irradiation and after ACP stimulation as determined by real-time PCR (**a**). Protein levels of Mstn after FL irradiation as measured by Western blotting (**b**). The levels of *Mstn* gene expression and protein were normalized to the signal intensity of the α-tubulin as the internal control. *P < 0.05; *NS*, not significant

FL irradiation of the skeletal muscle suppressed *Mstn* gene expression (Fig. 2a). Because suppression of gene expression is not correlated with a reduction in the amount of protein, we studied the amount of Mstn protein by using Western blotting. As Fig. 2b shows, the FL group had a significantly decreased Mstn protein level compared with the controls.

**Fig. 3:**
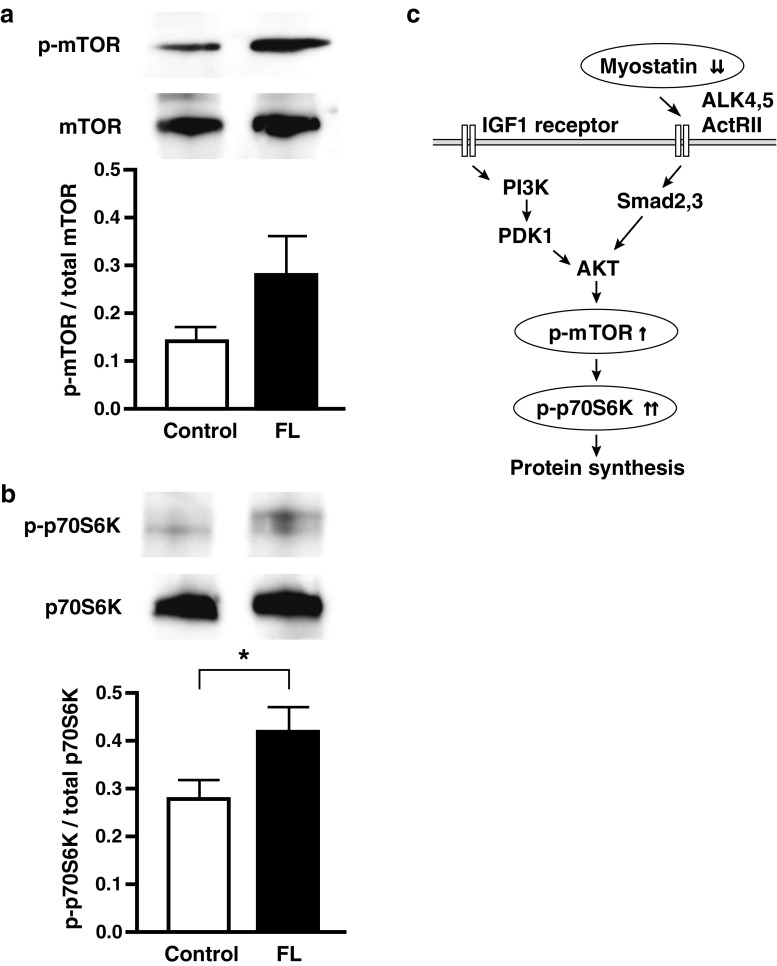
Phosphorylation of mTOR and p70S6K in skeletal muscle and schematic illustration of FL efficacy. Phosphorylation of mTOR (**a**) and p70S6K (**b**) after FL irradiation was analyzed via Western blotting. Summary of the efficacy of FL irradiation for protein synthesis (**c**). *One arrow*, increase or decrease; *two arrows*, significant increase or decrease. *IGF1* insulin-like growth factor 1, *ActRII* activin receptor II, *ALK* activin receptor-like kinase, *PI3K* phosphatidylinositol 3-kinase, *PDK1* 3-phosphoinositide-dependent protein kinase 1. *P < 0.05

To examine the influence of the reduced Mstn protein on the mTOR/S6K pathway, we investigated the activation of mTOR and p70S6K in the skeletal muscle after FL irradiation. Phosphorylation of mTOR tended to increase after FL irradiation, but the increase was not significant when compared with that of the control group (Fig. 3a). Phosphorylation of p70S6K significantly increased in the FL group (Fig. 3b).

## Discussion

In this study, we investigated the efficacy of FL irradiation of mouse skeletal muscle. As a result, we showed the reduction of amount of Mstn protein and the elevation of phosphorylation of p70S6K in mTOR/S6K signaling pathway by using Western blotting of the skeletal muscle.

In this study, our analysis here demonstrated significant reduction of *Mstn* gene expression in both FL and ACP groups compared with the control group (Fig. 2a), which was consistent with our previous data [11]. We investigated the Mstn protein level, which significantly decreased in skeletal muscles after FL irradiation, as Fig. 2b shows. In view of these results, FL irradiation suppressed both *Mstn* gene expression and Mstn protein level just as well as acupuncture treatment did. Furthermore, because of *Mstn* gene suppression, FL irradiation should be effective for a satellite cell-related proliferative reaction, such as seen with EA in our previous report [9].

To elucidate the involvement of FL in the mTOR/S6K signaling pathway, we examined the phosphorylation of mTOR and p70S6K after FL irradiation. mTOR phosphorylation tended to be elevated and p70S6K phosphorylation significantly increased after the irradiation (Figs. 3a, b). The increased phosphorylation of p70S6K indicates increased protein synthesis. Thus, our results suggested that FL irradiation activated protein synthesis. Figure 3c illustrates signal transduction after the irradiation. In a report describing acupuncture plus low-frequency electrical stimulation, that treatment upregulated protein synthesis-related proteins such as mTOR and p70S6K in skeletal muscles of diabetic mice [15]. Chronic low-frequency soleus electrostimulation during hindlimb unloading also significantly increased p70S6K phosphorylation in a rat model [16]. These reports and our results together suggest that the efficacy of FL irradiation is the same as that of acupuncture therapy or electrostimulation of skeletal muscle.

**Fig. 4:**
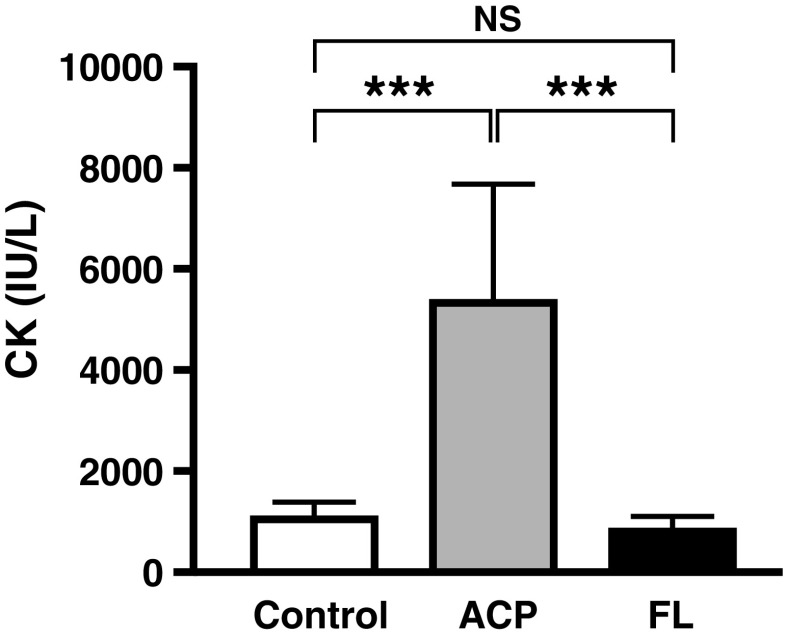
Serum CK after FL irradiation or ACP stimulation. ***P < 0.001; *NS*, not significant. Adapted from Takaoka et al. [11]

We previously measured serum creatine kinase (CK) activity to study the damage to skeletal muscle by FL irradiation [11]. Our results showed no difference in the CK activity levels between the FL and control groups, but the CK level in the ACP group, which had acupuncture needles inserted into skeletal muscle, was significantly greater than that in the other two groups (Fig. 4). The damage caused by FL irradiation was observed in the epidermis and dermis but did not reach the skeletal muscle, according to histological analysis [4, 11]. The depth of the damaged area was approximately 1100 μm. These results demonstrated that FLirradiation, in contrast to ACP stimuli, did not injure the skeletal muscle.

In summary, we reported here that FL irradiation of the skeletal muscle significantly reduced the amount of Mstn protein and enhanced the phosphorylation of p70S6K in the mTOR/S6K signaling pathway. Our results suggest that FL facilitated the activation of p70S6K, which led to promotion of the protein synthetic pathway. Our study here also suggests that FL irradiation can serve as an alternative to acupuncture needles. Compared with acupuncture with needles, FL acupuncture is non-invasive and carries no risk of infection. In addition, it can produce multipoint stimulation in a short time period. Therefore, additional detailed investigations of FL acupuncture are warranted.
